# Radiographic assessment of the femorotibial joint of the CCLT rabbit experimental model of osteoarthritis

**DOI:** 10.1186/1471-2342-10-3

**Published:** 2010-01-20

**Authors:** Caroline B Boulocher, Eric R Viguier, Rodrigo Da Rocha Cararo, Didier J Fau, Fabien Arnault, Fabien Collard, Pierre A Maitre, Olivier Roualdes, Marie-Eve Duclos, Eric P Vignon, Thierry W Roger

**Affiliations:** 1 Université de Lyon, Université Claude Bernard Lyon 1, UPSP 2007.03.135 RTI2B, Lyon, France; 2 École Nationale Vétérinaire de Lyon (ENVL), membre de l'université de Lyon, Lyon, France; 3 Hospices Civils Lyon Sud, Lyon, France

## Abstract

**Background:**

The purposes of the study were to determine the relevance and validity of in vivo non-invasive radiographic assessment of the CCLT (Cranial Cruciate Ligament Transection) rabbit model of osteoarthritis (OA) and to estimate the pertinence, reliability and reproducibility of a radiographic OA (ROA) grading scale and associated radiographic atlas.

**Methods:**

In vivo non-invasive extended non weight-bearing radiography of the rabbit femorotibial joint was standardized. Two hundred and fifty radiographs from control and CCLT rabbits up to five months after surgery were reviewed by three readers. They subsequently constructed an original semi-quantitative grading scale as well as an illustrative atlas of individual ROA feature for the medial compartment. To measure agreements, five readers independently scored the same radiographic sample using this atlas and three of them performed a second reading. To evaluate the pertinence of the ROA grading scale, ROA results were compared with gross examination in forty operated and ten control rabbits.

**Results:**

Radiographic osteophytes of medial femoral condyles and medial tibial condyles were scored on a four point scale and dichotomously for osteophytes of medial fabella. Medial joint space width was scored as normal, reduced or absent. Each ROA features was well correlated with gross examination (p < 0.001). ICCs of each ROA features demonstrated excellent agreement between readers and within reading. Global ROA score gave the highest ICCs value for between (ICC 0.93; CI 0.90-0.96) and within (ICC ranged from 0.94 to 0.96) observer agreements. Among all individual ROA features, medial joint space width scoring gave the highest overall reliability and reproducibility and was correlated with both meniscal and cartilage macroscopic lesions (r_s _= 0.68 and r_s _= 0.58, p < 0.001 respectively). Radiographic osteophytes of the medial femoral condyle gave the lowest agreements while being well correlated with the macroscopic osteophytes (r_s _= 0.64, p < 0.001).

**Conclusion:**

Non-invasive in vivo radiography of the rabbit femorotibial joint is feasible, relevant and allows a reproducible grading of experimentally induced OA lesion. The radiographic grading scale and atlas presented could be used as a template for in vivo non invasive grading of ROA in preclinical studies and could allow future comparisons between studies.

## Background

Osteoarthritis (OA) is a progressive disorder of the joints caused by gradual loss of cartilage. OA is also known as degenerative joint disease. OA is a painful chronic disease of the synovial joints. It is the major source of disability in the elderly population impairing their ability to perform many activities of daily living. The prevalence of OA is increasing with the aging of Western populations [1-11]. Non invasive procedures such as MRI, ultrasonography and laboratory biomarkers are gaining wider acceptance in clinical studies [12-15]. However according to regulatory requirements radiographic OA quantification remains the most relevant evaluation of OA natural progression [16-19]. Standard radiographic atlas and grading systems are recommended for radiographic assessment of human OA in both clinical and epidemiological trials of OA natural progression and disease-modifying osteoarthritis agents (DMOA's) therapeutic evaluation [17,20-25]. Several grading system have been created and evaluated in knee, hip, hand and foot ROA studies and are illustrated in atlases [26-35].

Studies in animal experimental models of OA provide a broad spectrum of outcome parameters. They are used to elucidate the OA development process and the mechanisms responsible for its progression leading to the discovery of potential therapeutic targets. In particular, the cranial cruciate ligament transection (CCLT) of the rabbit femorotibial joint is a well accepted surgically induced model of OA in the development of DMOA strategies [36-42]. Qualitative evaluation of OA and a few quantitative rating scales are described in the equine and canine [43-48]. Reports on radiographic evaluation of the rabbit femorotibial joint are scarce and to our knowledge, no standardized in vivo non-invasive radiographic protocol and no atlas of individual ROA features associated with a radiographic grading scale have yet been described to evaluate in vivo ROA progression in the CCLT rabbit model [49-51]. Information on both intra- and inter-observer agreements is fundamental to the development of an effective scoring system and needs to be estimated prior to its implementation [52,53].

We believe such atlas and grading ROA scale would add benefits to radiography as an outcome measure in longitudinal and cross sectional in vivo experimental OA studies. The first aim of the study was to validate a composite ROA grading scale of the femorotibial joint of the CCLT rabbit experimental model of OA. Semi-quantitative ROA scale was illustrated in a radiographic atlas of individual radiographic features of OA. The second aim of the study was to estimate inter and intraobserver agreement.

## Methods

All work was conducted in accordance with the Ethical Committee Guidelines of the Ecole Vétérinaire de Lyon (ENVL, France).

### Animal procedures

#### Experimental OA induction

During previous studies experimental OA was induced surgically by CCLT in the left femorotibial joint of New Zealand White rabbits while the right joint was left intact. These rabbits formed the operated group. Rabbits of the control group were not operated on. Rabbits were kept in individual cages and in the same conditions [40,50,51,54].

#### Radiographic protocol

**Figure 1:**
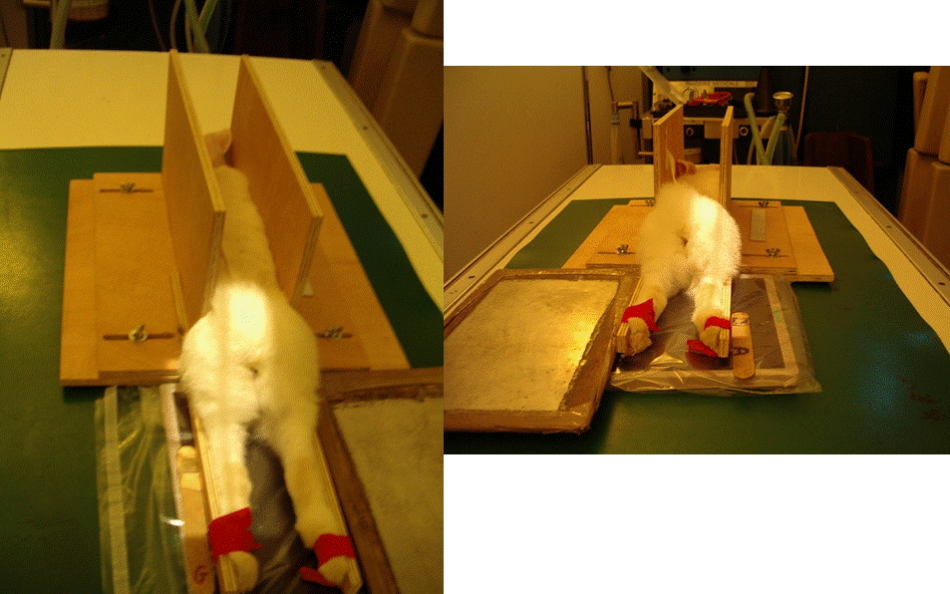
**Rabbit positioning for the extended caudo-cranial non weight-bearing radiography of the knee joint**. Sedated rabbits were placed in sternal recumbency with both leg extended caudally and individually fitted with an elastic bandage to the dedicated extensions of the "radiographic rabbit bed"

Rabbit positioning is illustrated in Figure 1. In vivo non-invasive radiographs of the femorotibial joints were performed prior to the surgery and monthly up to five months in both control and operated groups in a non weight-bearing extended caudo-cranial view. Radiography was performed by the same operator with the same equipment (46 kV, 200 mA, 32 ms, Trophy N800 HF, Fujifilm 24*30 cm^2 ^IP cassette type C, 1 m film-focus distance). Rabbits were sedated by intramuscular injection of xylazine (Rompun 2%^®^, Bayer 3 mg/kg) and ketamine (Imalgène 1000^®^, Merial, 30 mg/kg). Sedated rabbits were placed in sternal recumbency with both legs extended caudally and individually fitted with an elastic bandage to the dedicated extensions of a wooden customized "radiographic rabbit bed"(figure 1). The width between the bed sides and extensions was adjusted to the rabbit pelvic width so that both legs were parallel to the rabbit sagittal plane. Radiographs were made with a vertical X-ray beam centered over the femorotibial joints and collimated from the mid femur to the mid tibia.

### Film evaluation

#### Radiographic OA scoring and composite ROA atlas creation

To determine the feasibility of a radiographic grading system, three experienced readers reviewed 250 radiographs of rabbit left femorotibial joint from control and CCLT rabbits obtained during previous studies [40,50,51,54]. Subsequently they consensually constructed an original ROA semi-quantitative grading scale that separated joint space narrowing and osteophytosis [35].

**Figure 2:**
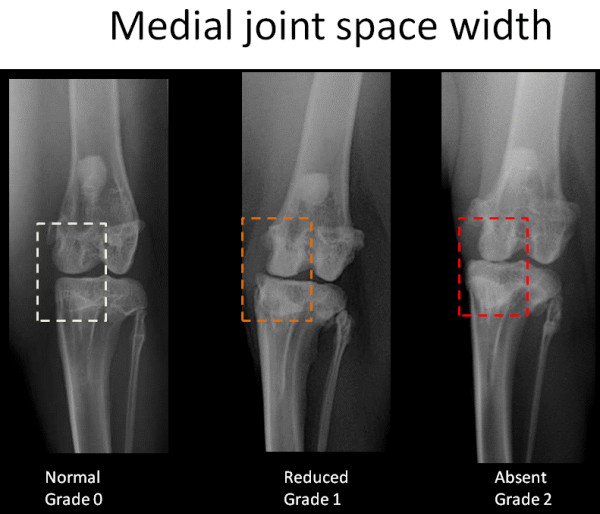
**Radiographic atlas of individual OA features in the CCLT rabbit model of OA**. Medial joint space width: normal (grade 0), reduced (grade 1), absent (bone to bone)(grade 2)

**Figure 3:**
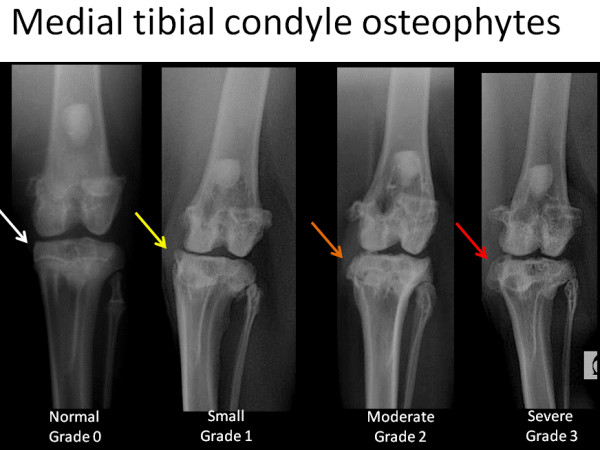
**Radiographic atlas of individual OA features in the CCLT rabbit model of OA**. Medial tibial condyles osteophytes: absent (grade 0), small (grade 1), moderate (grade 2), severe (grade 3)

**Figure 4:**
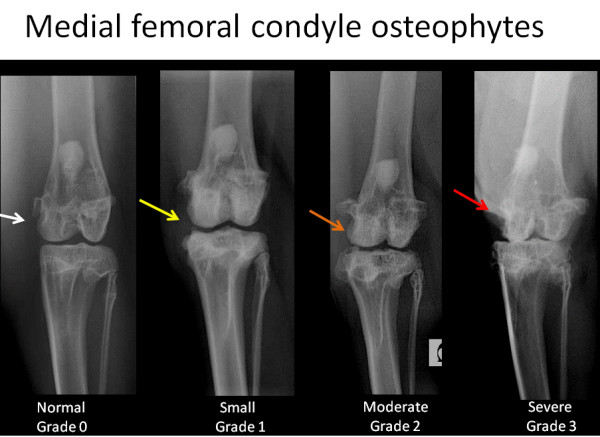
**Radiographic atlas of individual OA features in the CCLT rabbit model of OA**. Medial femoral condyles osteophytes: absent (grade 0), small (grade 1), moderate (grade 2), severe (grade 3)

**Figure 5:**
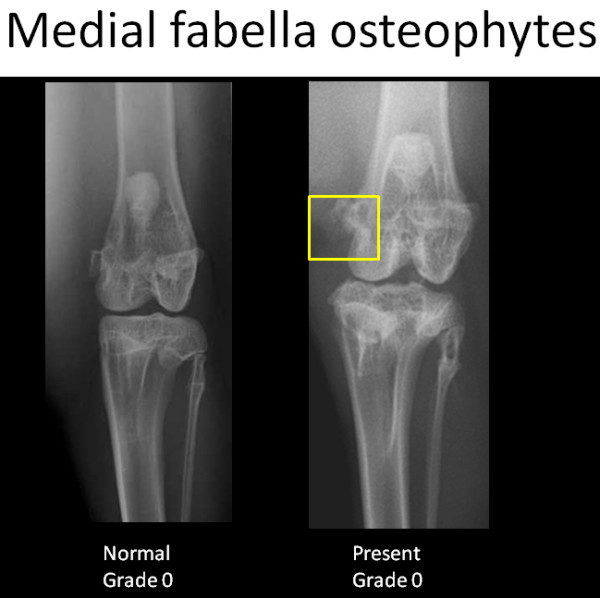
**Radiographic atlas of individual OA features in the CCLT rabbit model of OA**. Medial fabella osteophytes: absent (grade 0), present (grade 1)

To create the corresponding radiographic atlas, the same three readers consensually chose a representative digital radiograph for each radiographic score of each individual ROA feature. Femorotibial medial joint space width was graded as normal (grade 0), reduced (grade 1) or absent ie. bone to bone (grade 2). Osteophytes of the medial femoral condyle and osteophytes of the medial tibial condyle were scored separately according to their presence and size (grade 0 = absence, grade 1 = small, grade 2 = moderate, grade3 = severe). Osteophytes of the medial fabella were scored dichotomously as absent or present (grade 0 or grade 1, respectively). The radiographic atlas is presented in Figures 2, 3, 4 and 5.

#### Reading procedures and reproducibility

The first author selected a sample of fifty radiographs representing a wide range of ROA changes with optimized femorotibial joint positioning and adequate quality.

- Optimized femorotibial joint position was defined as: midline of the patella within the femoral trochlear groove superimposed on the femoral long axis and aligned with the trochlear notch; medial and lateral femoral condyles symmetrical to the femoral long axis; and fibulo-tibial joint space clearly outlined.

##### - Interobserver agreement

Radiographs of the left femorotibial joint from control and operated rabbits were presented on random order and the dates of exam and rabbit status were hidden. Readers were instructed to grade the femorotibial joint by reference to the radiographic atlas of the individual radiographic features. Five readers (veterinary surgeons) interpreted films independently, without knowledge of each other's results and blind to the femorotibial joint status and identity (operated *vs *control, date of surgery). The first author did not perform the film reading.

##### - Intraobserver agreement

Three of the five readers separately repeated the rating with a time interval of four weeks between the two readings and without knowledge of previous results.

##### - Time efficiency

Readers were asked to report to the first author the time spent for each reading sessions.

### Macroscopic and radiographic correlation at 5 months

After radiographic examination at 5 months, forty operated and ten control sedated rabbits were euthanized by intra-cardiac injection of 3 mL of pentobarbital sodium. Macroscopic and radiographic scores resulted from a consensual grading performed by three of the authors. Gross examination and ROA grading were done independently then compared.

Meniscal injuries were reported in a semi-quantitative grading table modified from the human literature. Gross morphological cartilage changes were evaluated using Visual Analogic Evaluation (VAE) for the articular surface of the tibia and of the femur. This score is based on the International Cartilage Repair Society (ICRS) recommendations for grading cartilage defects. The VAE score results from the product of the percentage of the area involved and a factor based on the grade of the cartilage lesion. Grades were assessed by noting the most advanced lesion present within the cartilage, irrespective of its horizontal extent (grade = depth of cartilage erosion). The VAE score ranges from 0 indicating that the cartilage is intact to 100 meaning that full-thickness cartilage erosion occurred [50,51,54,55].

### Data analysis

Student's paired tests and non parametric spearman rank correlation coefficient (r_s_) were used to evaluate the pertinence of the radiographic grading scale and compare macroscopic and ROA results. A p < 0.05 was considered statistically significant.

Agreements were estimated with Intra-class coefficients (ICCs) and their corresponding 95% confidence intervals (CIs). ICCs values near zero indicate imperfect reliability of the grading scale while values near 1.0 indicate perfect reproducibility. ICCs were interpreted as follows: poor agreement below 0.40, fair to good from 0.40 to 0.75, and excellent above 0.75. Both single-measure ICC and average-measure ICC methods were calculated as further research design might involve either one or an average of readers' grading. Consistency type of ICC was calculated based on a two-way random effects model to allow generalization of the results to all possible readers. Inter-observer agreement was computed with the five readers first reading session results for each individual OA features, total osteophytes and global OA scores. Intraobserver agreement was similarly assessed for each individual OA features, total osteophytes and global OA scores. In addition absolute type ICC was used for each individual observer to provide the range of reliability of the scale between different readers [56-58].

Statistical analyses were undertaken with SPSS for windows, version 15.0 (SPSS, Chicago, IL).

## Results

### Pertinence of the radiographic grading scale

#### Macroscopic OA lesions [50,51]

Five months after surgery, gross examination showed intact CCL in the femorotibial joint of control rabbits. In all of the operated joints, the CCLT was completely transected and associated with severe changes consistent with the development of chronic OA. Operated joints exhibited femoral and tibial chondropathy from oedema to full thickness cartilage erosion and bone ulceration; severe degradation of the menisci; extensive tibial and femoral condyles remodeling; soft tissue fibrosis and osteophytosis.

#### ROA composite grading scale of the medial compartment

The scoring system is described in Table 1 and is illustrated by the radiographic atlas in Figures 2, 3, 4 and 5.

**Table 1:** Radiographic grading scale

Radiographic OA feature of the medial compartment		Grade 0	Grade 1	Grade 2	Grade 3
**Joint space width**		**Normal**	**Reduced**	**Absent**	**NA**

Osteophytes	Medial tibial condyle	Absent	Small	Moderate	Severe
	
	Medial femoral condyle	Absent	Small	Moderate	Severe
	
	Medial fabella	Absent	Present		NA

Total osteophytes				0-7	

Global ROA score				0-9	

Osteophytes of the medial compartment corresponded to the aggregate of the osteophytes (range 0-7). Global ROA score was obtained by the summation of the individual feature grades and ranged from 0 to 9.

### Macroscopic and radiographic correlation at 5 months [50,51]

Final macroscopic and radiographic OA lesion evaluation were significantly positively correlated for the medial compartment (p < 0.001). Radiographic scores of each individual ROA lesion were significantly higher in the operated group than in the control group (p < 0.001).

Macroscopic and radiographic osteophytes were well correlated (r_s _= 0.64, p < 0.001). Joint space narrowing (JSN) was correlated with macroscopic meniscal lesions (r_s _= 0.68, p < 0.001) and with tibial cartilage lesions (r_s _= 0.58, p < 0.001). Medial tibial cartilage lesions score were significantly higher in rabbits with a narrowed or absent medial joint space (JSN grade of 1 or 2) than in rabbits with normal joint space width, respectively p < 0.05 and p < 0.001. Medial tibial cartilage lesions were lower but not significant statistically in rabbits with a narrowed JS than in rabbits with an absent JS (grade of 1 and 2), p > 0.05.

### Evaluation of the radiographic atlas validity

#### Time efficiency

The average time required to read the fifty radiographs was two hours.

#### Range of ROA lesions

Results are shown in Table 2. Results from first reading session of the most consistent reader were used to evaluate the distribution of the individual ROA features. The total radiographic score ranged from 0 to 9 meaning the full range of radiological features of osteoarthritis of the femorotibial joint in the rabbit CCLT experimental model was available for the evaluation for the atlas.

**Table 2:** Descriptive analyses (number of sample = 50)

Descriptive statistics	Min	Max	Mean	*SD*
Medial joint space narrowing	0	2	0.86	0.80

Osteophytes of the medial tibial condyle	0	3	1.2	1.0

Osteophytes of the medial femoral condyle	0	3	0.7	0.82

Osteophytes of the medial fabella	0	1	0.6	0.50

Global OA score	0	9	3.4	2.90

#### Interobserver agreement

Results are detailed in Table 3. Average-measures ICCs were always higher than single-measures ICCs which are detailed here. Inter-observer agreement for the osteophytes of the medial compartment was excellent, with ICCs value of (ICC 0.90; CI 0.85-0.94). Global ROA score ICC value also demonstrated excellent reproducibility (ICC 0.93; CI 0.90-0.96) Interobserver agreement for the medial joint space width was the highest of the individual ROA markers (ICC 0.91; CI 0.87-0.94) and was the lowest for the osteophytes of the medial fabella (ICC 0.74; CI 0.64-0.83). Inter-observers ICCs for the osteophytes of the medial tibial condyle was slightly higher than for the medial femoral condyle (respectively ICC 0.76 with CI 0.66-0.84 and ICC 0.88 with CI 0.83-0.92).

**Table 3:** Inter-observer agreement

Inter-observer agreement (Consistency type ICC- Five readers)								
	**Medial JSW**		**Osteophytes of the medial compartment**					
			
**ROA features**			**Medial tibial Condyle**		**Medial femoral condyle**		**Medial fabella**	
	
	**ICC**	**CI**	**ICC**	**CI**	**ICC**	**CI**	**ICC**	**CI**

Single-measure	0.91	0.87-0.94	0.88	0.83-0.92	0.76	0.66-0.84	0.74	0.64-0.83

Average-measure	0.98	0.97-0.98	0.97	0.96-0.98	0.94	0.91-0.96	0.93	0.90-0.96

**Inter-observer agreement (Consistency type ICC- Five readers)**								
				
**ROA features**	**Medial Osteophytes**		**Total OA**					
					
	**ICC**	**CI**	**ICC**	**CI**				
				
Single-measure	0.90	0.85-0.94	0.93	0.90-0.96				
				
Average-measure	0.98	0.97-0.99	0.99	0.98-1.0				

#### Intraobserver agreement

The highest reliability was seen for the medial joint space width (ICC ranged from 0.94 to 1.0). Global ROA score gave the highest reliability (ICC ranged from 0.94 to 0.96). The lowest reliability was observed for the osteophytes of the medial femoral condyle which was only moderate for one reader (ICC 0.70) but still good for the other two readers (ICCs 0.79 and 0.90).

## Discussion

Radiography is the least expensive method of imaging joints and is more readily available than MRI. In man, radiographic OA is defined on the evaluation of joint space width (JSW) and osteophytes [1-8,21,22]. Severity of radiographic OA can be estimated using semi-quantitative scoring systems. For nearly half-century the Kellgren and Lawrence system has extensively been used in large clinical and epidemiological studies. However, such a global assessment is invalid as it assumes that changes in radiographic features are linear over the course of the disease with constant relationship. Clinical studies and research on OA pathogenesis require separate assessment of the different OA features [9,10,16,19,59-69]. To overcome this problem, Altman and others developed a radiographic grading scale of individual OA features. This scale is illustrated in a radiographic atlas which has been updated in 2007 and published on line by the Osteoarthritis Research Society International (OARSI) [35].

To the authors' best knowledge, prior to this report, while being well characterized by histology and by gross examination in the literature no radiographic atlas of the CCLT rabbit model of OA has been described. Equally neither recommendations nor detailed description of the radiographic procedure could be found. In man JSN and osteophyte scoring provide better reproducibility for grading ROA than cyst, sclerosis or bone contour. We based the CCLT rabbit model composite ROA atlas and grading scale on osteophyte and JSN grading. ICCs of each individual ROA features demonstrated excellent agreement inter- and intra-readings. Global ROA score gave the highest ICCs value for both between and within observer agreements. In all individual ROA features, JS narrowing gave the highest overall reliability and reproducibility.

The simple "rabbit bed" permitted easy standardization of the fully extended caudocranial view of the rabbit femorotibial joint, with both rabbit metatarsi parallel to the sagittal plane of the animal, but precluding weight-bearing. We are currently working on a dedicated system to perform in vivo weight-bearing radiographs of the rabbit femorotibial joints.

Poor radio-anatomic positioning is known to be an important source of error of JSW (and JSN) evaluation. Manually applied stress on non weight-bearing caudocranial radiographs of the canine femorotibial joint, has the most marked effect on the JSW [70]. In contrast, mild to moderate decentering of the Xray beam along the long or transversal axes of the hind leg does not alter JSW as much as rotation or manually applied mediolateral stress. To limit positioning artifact we fixed the rabbit metatarsi to the bed extensions ensuring simple and reproducible positioning. As radiographic procedure was performed by the same person (CB) this also helped to reduce positioning errors. Further work is needed to evaluate the consequences of the OA changes on the positioning of the femorotibial joint for the ROA grading as OA impairs the joint range of motion.

In CCLT rabbit model of OA, the severity of the chondropathy is reported to vary widely between subjects and to be associated with meniscal lesions and it is not clear whether structural cartilage or meniscal lesions appear first in the CCLT rabbit. Worthy of notice, at five months after surgery ROA severity was positively correlated both with meniscal and cartilage macroscopic lesions. Further work is needed to distinguish the sequential development of these lesions [36-42].

Similarly to the CCLT canine experimental model of OA, the radiographic details of the operated rabbits' femorotibial joint allowed observation of central osteophyte and subchondral cystic lesions. Gross examination does not allow quantification of subchondral lesions. We are currently investigating histological analyses to integrate these features in a ROA grading scale.

Poor correlation has often been reported between macroscopic and histologic tibial cartilage thickness of the rabbit femorotibial joint [50,54]. This is due to the inherent problems of using a unidimensional measure (histology) to indirectly evaluate changes in 3D structures within a joint compartment.

Non-invasive surrogates such as tomographic imaging procedure (μCT, μMRI) still need to be validated in CCLT rabbit model of OA.

## Conclusion

In this study, we demonstrated radiographic semi-quantitative grading of individual OA features in the rabbit CCLT model of OA was feasible, relevant and reproducible. The standardized protocol and the radiographic atlas presented here could be used as a template for semi-quantitative grading of the ROA in preclinical studies.

## Abbreviations

CCLT: Cranial Cruciate Ligament Transection; CI: Coefficient Interval; DMOA: Disease Modifying Osteoarthritis Agent; ICC: Intraclass Correlation Coefficient; JSN: Joint Space Narrowing; JSW: Joint Space Width; μCT: micro-Computed Tomography; μMRI: micro- Magnetic Resonance Imaging; NA: Non Applicable; OA: Osteoarthritis; ROA: Radiographic Osteoarthritis.

## Competing interests

The authors declare that they have no competing interests.

## Authors' contributions

All authors read and approved the final manuscript. CB carried out the study design, the acquisition of all the radiographs, the analysis and interpretation of data and wrote the manuscript. TR, EV, EPV conceived the study, participated in its design and coordination, and helped to draft the manuscript. DF, FA, PM, FC, MED and OR were involved in the acquisition of the data. RC, EV, MED, DF performed the CCLT. CB, EV, TR, MED performed the gross examination. CB, EV, and TR conceived the radiographic atlas. CB, DF, FA, FC, PM read the radiographs.

## Pre-publication history

The pre-publication history for this paper can be accessed here:

http://www.biomedcentral.com/1471-2342/10/3/prepub
